# Supplementary material for: Whole-exome sequencing identifies protein-coding variants associated with brain iron in 29,828 individuals
Source: Nat Commun. 2024 Jul 2;15:5540. doi: 10.1038/s41467-024-49702-2 (PMC11219919; doi:10.1038/s41467-024-49702-2)
Supplement: Supplementary file 1 — Supplementary Information [file 41467_2024_49702_MOESM1_ESM.pdf]

Supplementary Material for “Whole-Exome Sequencing Identifies Protein-Coding Variants Associated with Brain Iron in 29,828 Individuals”

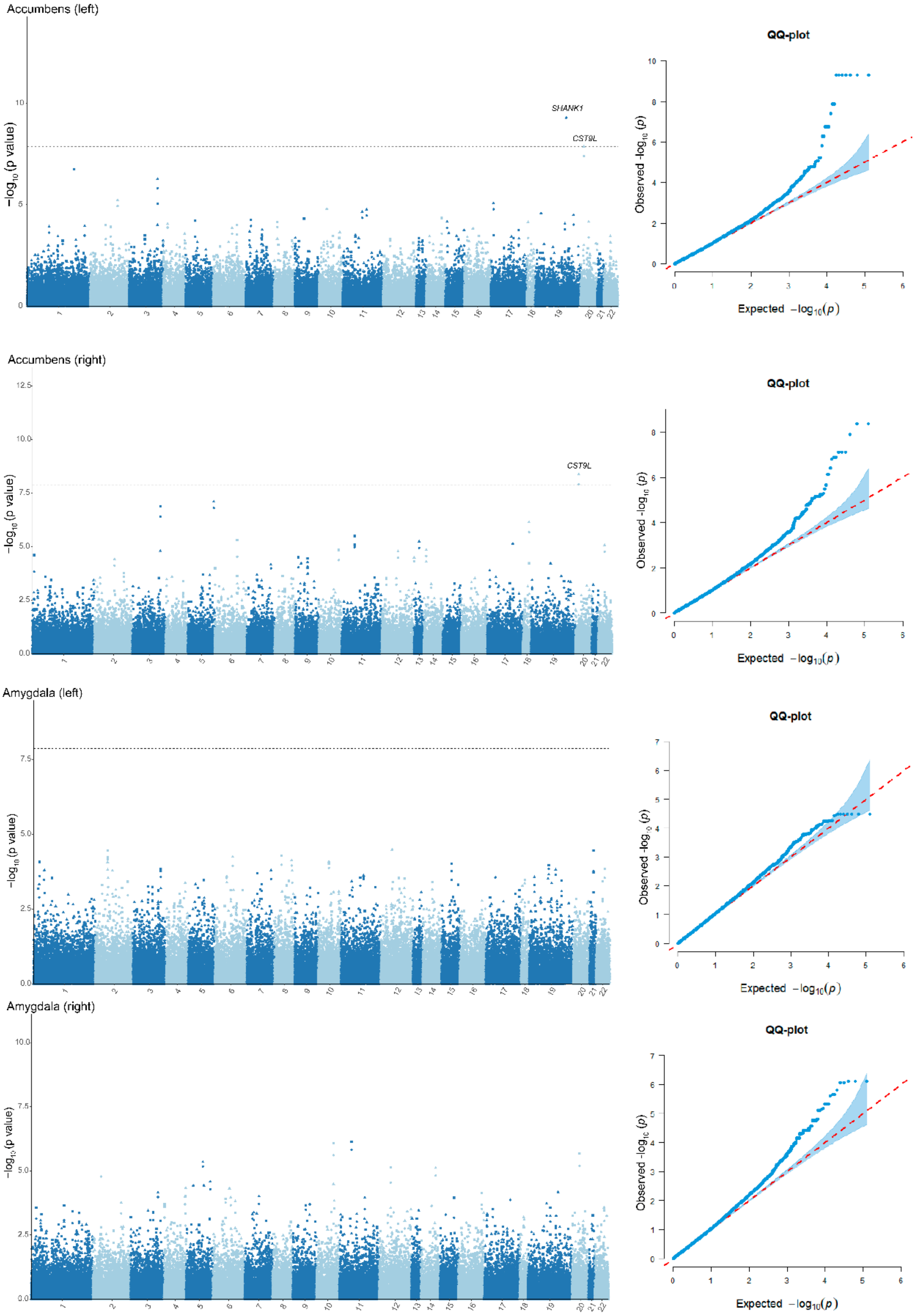

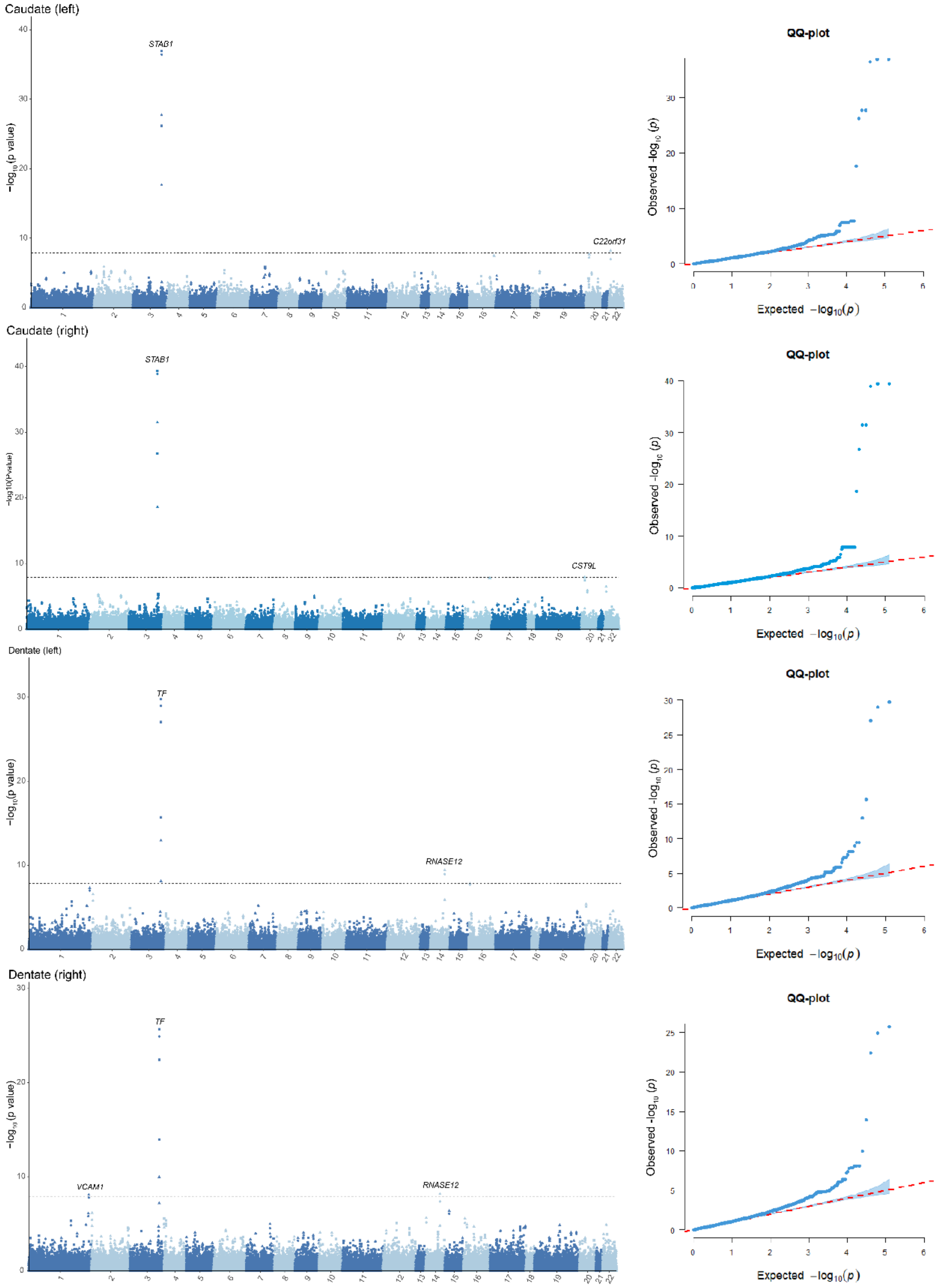

Caudate (left)
STAB1
C22orf31
−log10(p value)
QQ-plot
Observed -log10(p)
Expected −log10(p)
Caudate (right)
STAB1
CST9L
−log10(Pvalue)
QQ-plot
Observed -log10(p)
Expected −log10(p)
Dentate (left)
TF
RNASE12
−log10(p value)
QQ-plot
Observed -log10(p)
Expected −log10(p)
Dentate (right)
TF
VCAM1
RNASE12
−log10(p value)
QQ-plot
Observed -log10(p)
Expected −log10(p)

Globus pallidus externa (left)

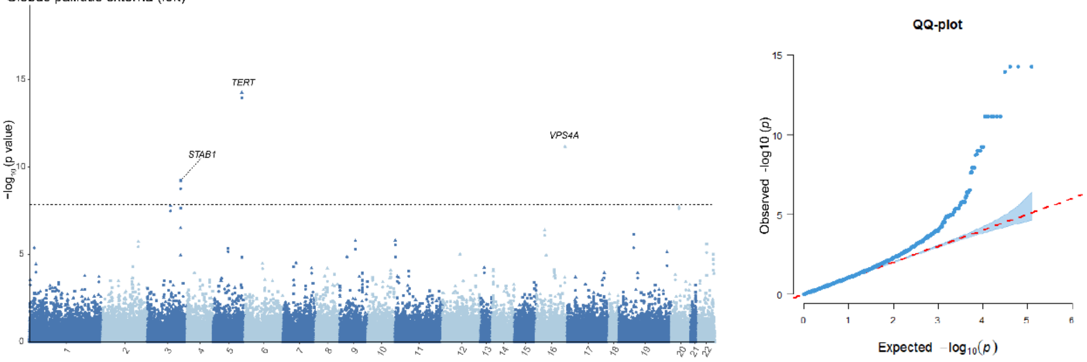

Globus pallidus externa (right)

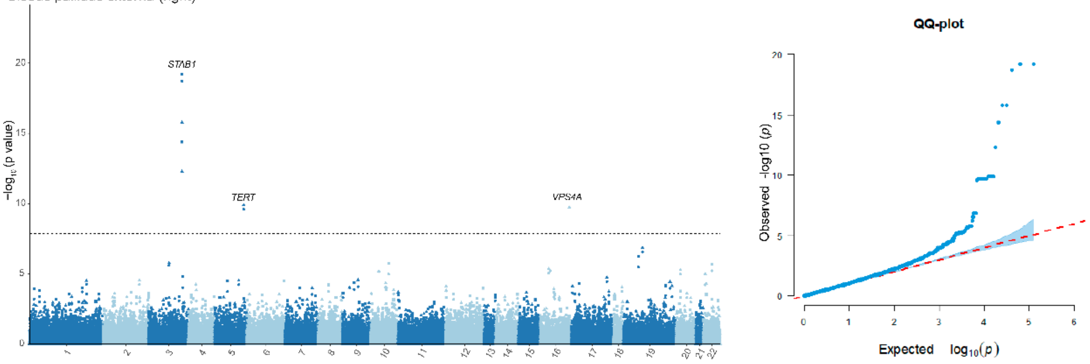

Globus pallidus interna (left)

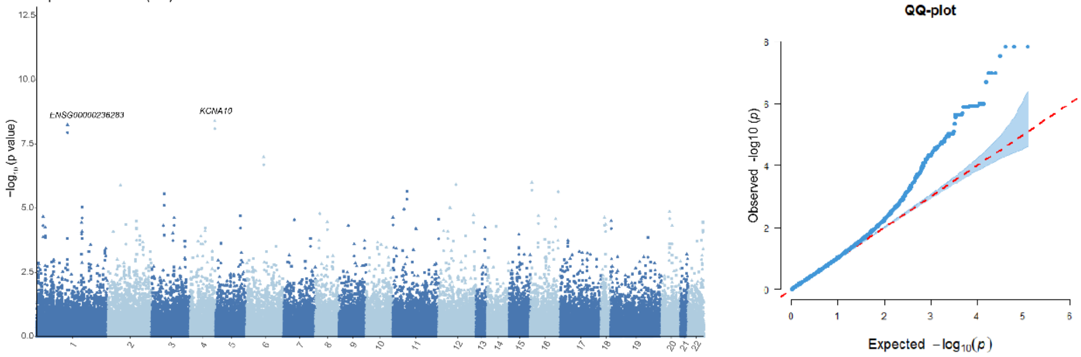

Globus pallidus interna (right)

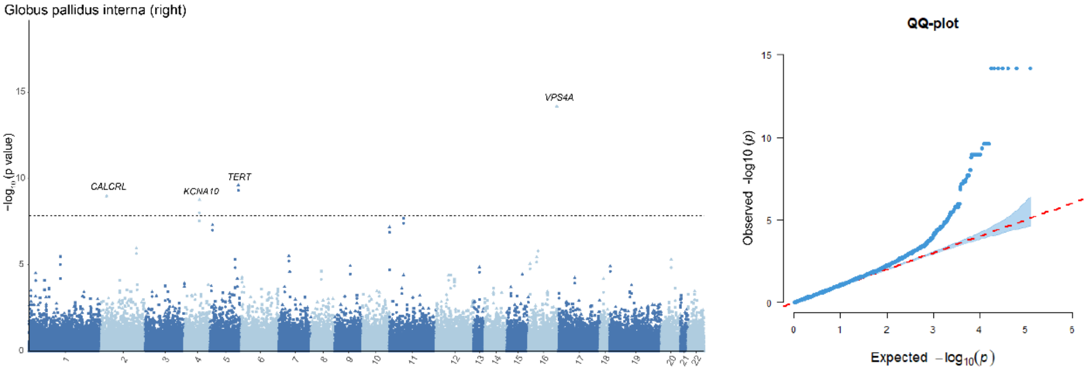

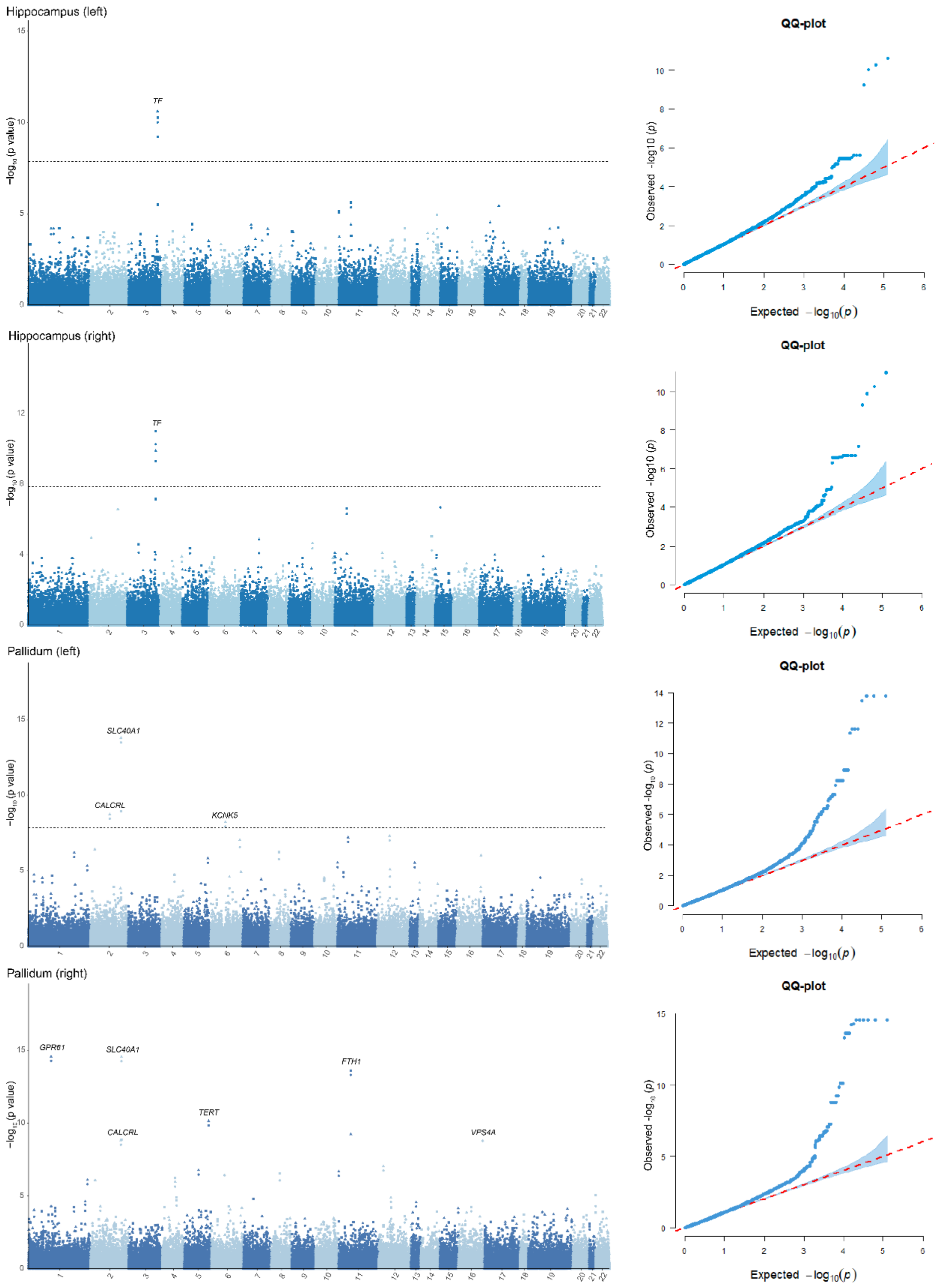

Hippocampus (left)
TF
QQ-plot
Hippocampus (right)
TF
QQ-plot
Pallidum (left)
SLC40A1
CALCRL
KCNK5
QQ-plot
Pallidum (right)
GPR61
SLC40A1
FTH1
TERT
CALCRL
VPS4A
QQ-plot

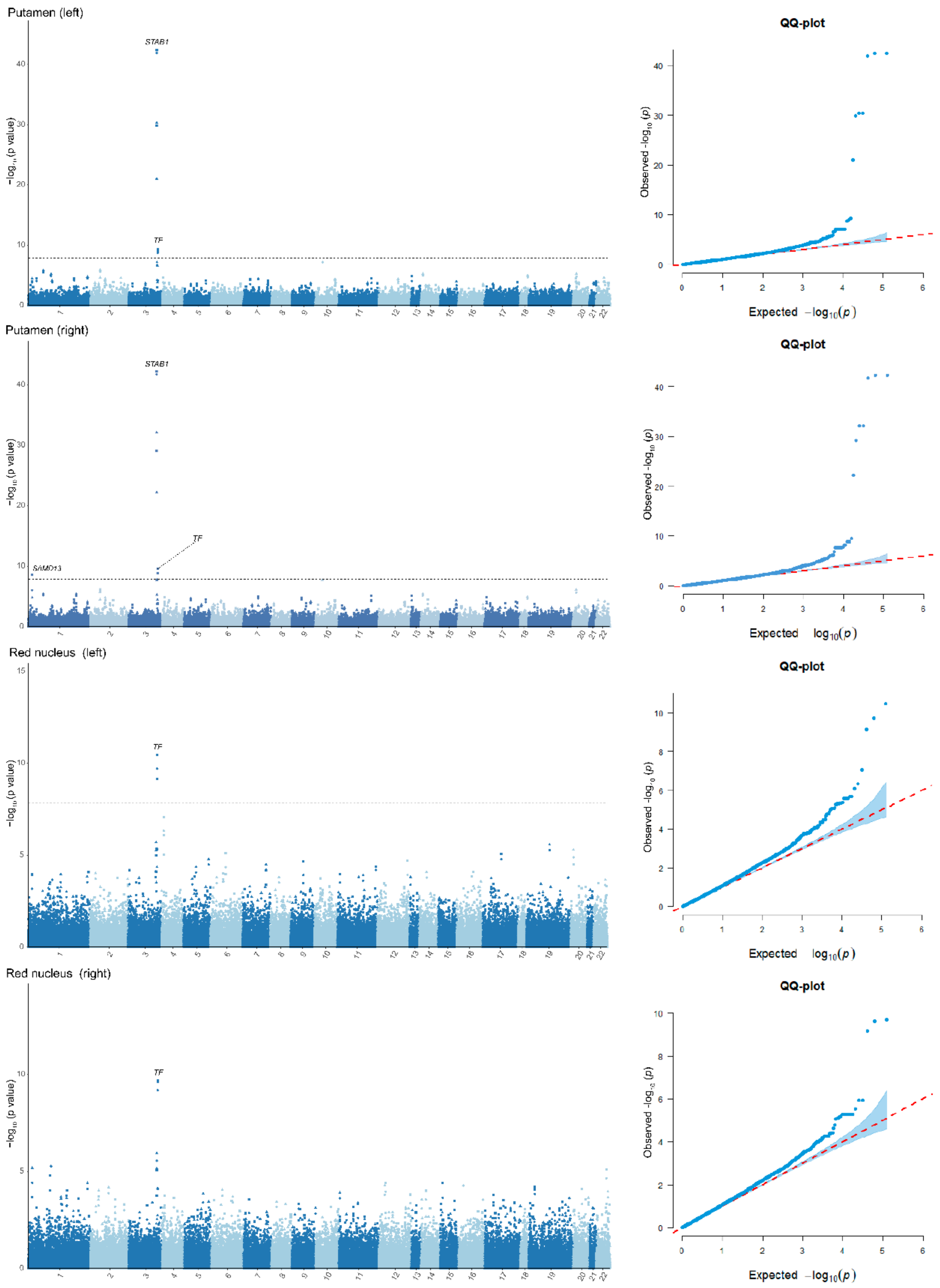
Putamen (left)
STAB1
TF
QQ-plot
Putamen (right)
STAB1
TF
SAMD13
QQ-plot
Red nucleus (left)
TF
QQ-plot
Red nucleus (right)
TF
QQ-plot
Observed -log10 (p)
Expected -log10(p)
-log10 (p value)

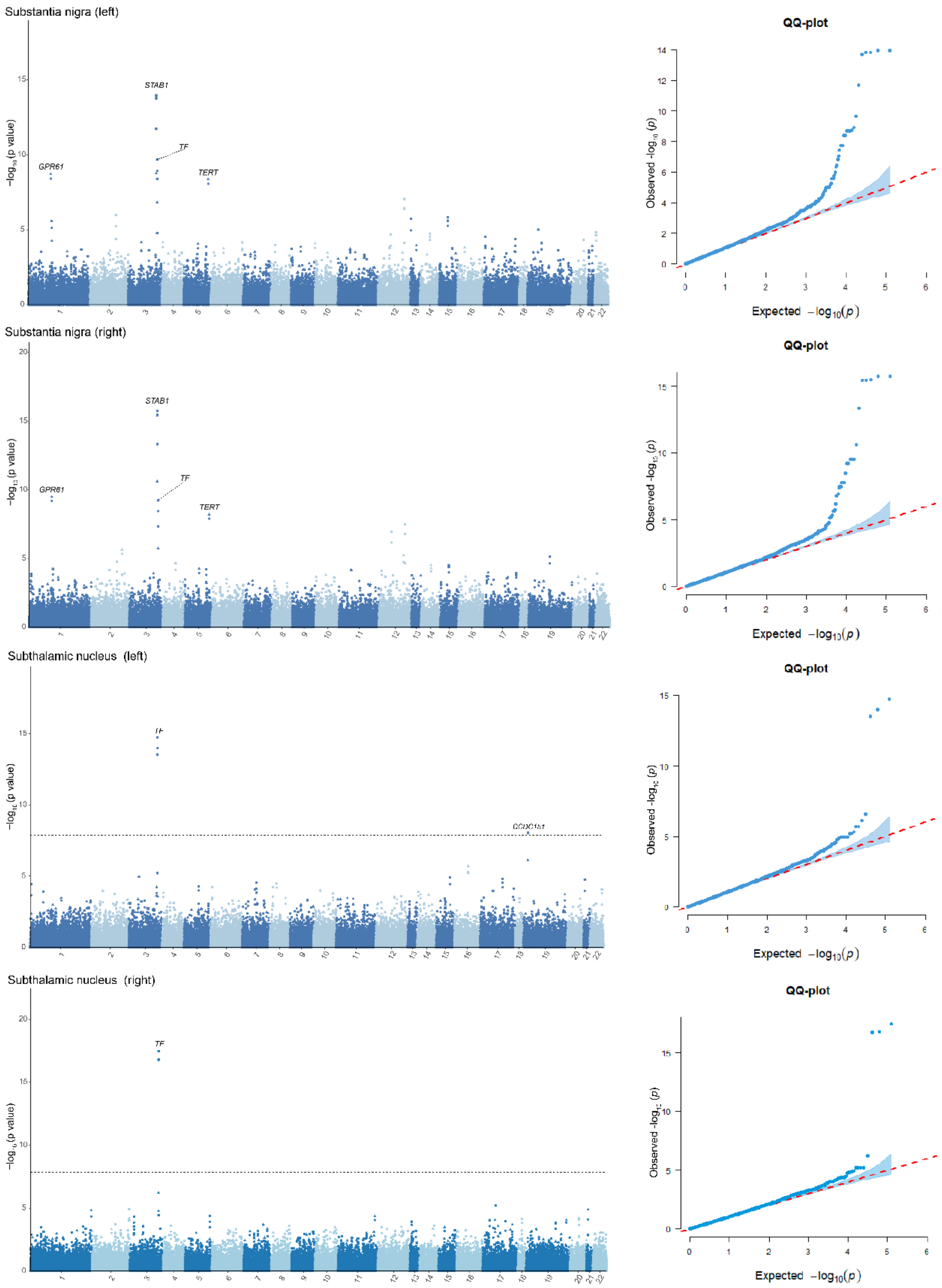

Substantia nigra (left)
STAB1
TF
GPR61
TERT
−log10 (p value)
QQ-plot
Observed -log10 (p)
Expected −log10(p)
Substantia nigra (right)
STAB1
TF
GPR61
TERT
−log10 (p value)
QQ-plot
Observed -log10 (p)
Expected −log10(p)
Subthalamic nucleus (left)
−log10 (p value)
QQ-plot
Observed -log10 (p)
Expected −log10(p)
Subthalamic nucleus (right)
TF
−log10 (p value)
QQ-plot
Observed -log10 (p)
Expected −log10(p)

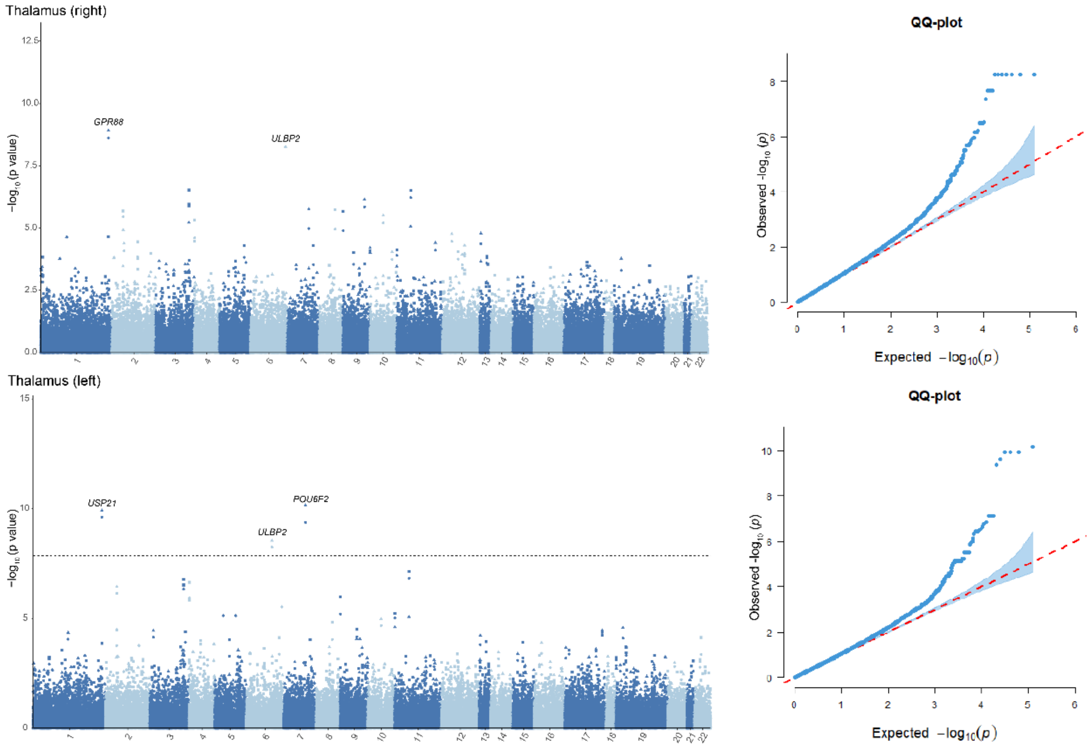

Supplementary Figure 1. Manhattan plots of every exome-wide association analysis of rare protein-coding variants performed for QSM in 26 brain regions. in 26789 participants. P values shown are two-sided and Bonferroni correction were used. The dotted horizontal line represents the significance threshold (1.7×10^{-8}).

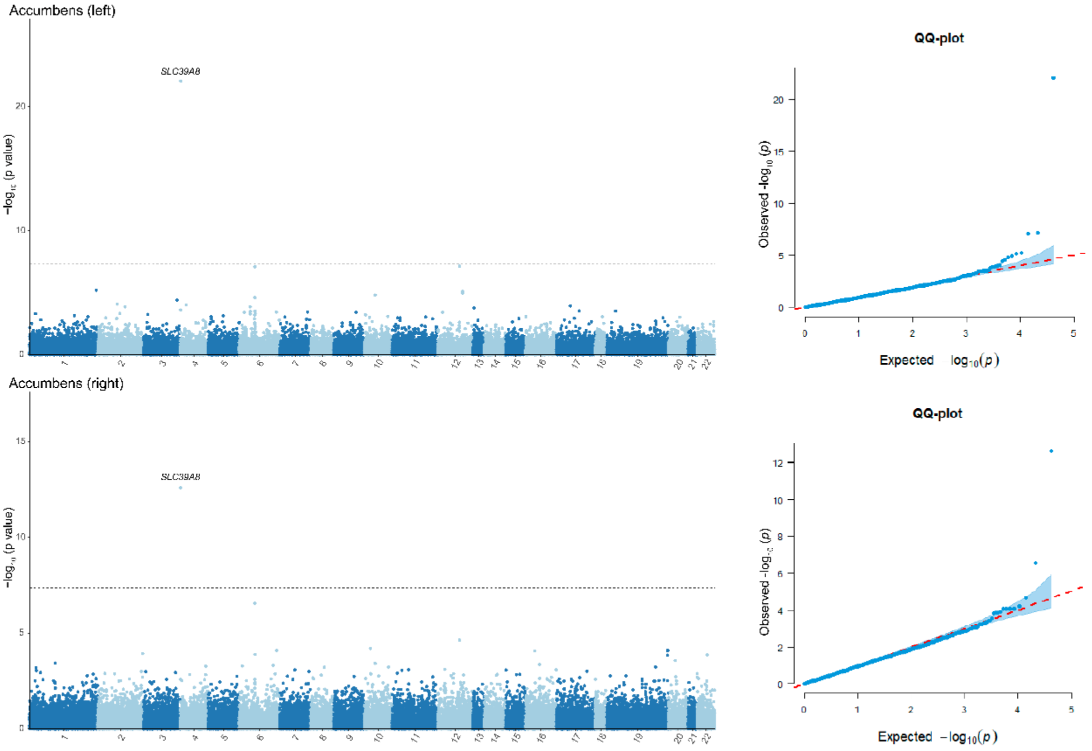

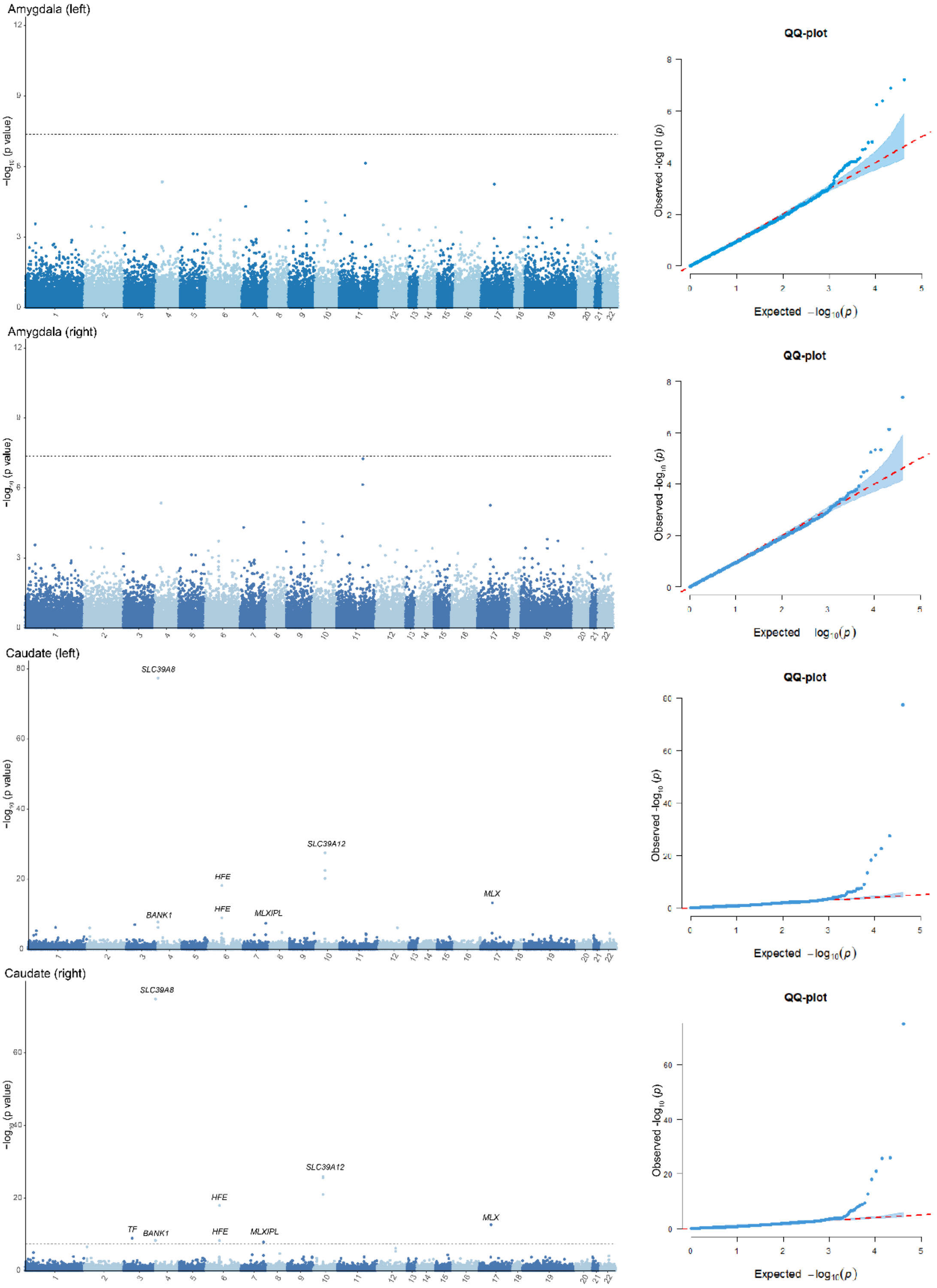
Amygdala (left)
QQ-plot
Amygdala (right)
QQ-plot
Caudate (left)
SLC39A8
SLC39A12
HFE
HFE
BANK1
MLXIPL
MLX
QQ-plot
Caudate (right)
SLC39A8
SLC39A12
HFE
TF
BANK1
HFE
MLXIPL
MLX
QQ-plot
-log10 (p value)
Observed -log10 (p)
Expected -log10(p)

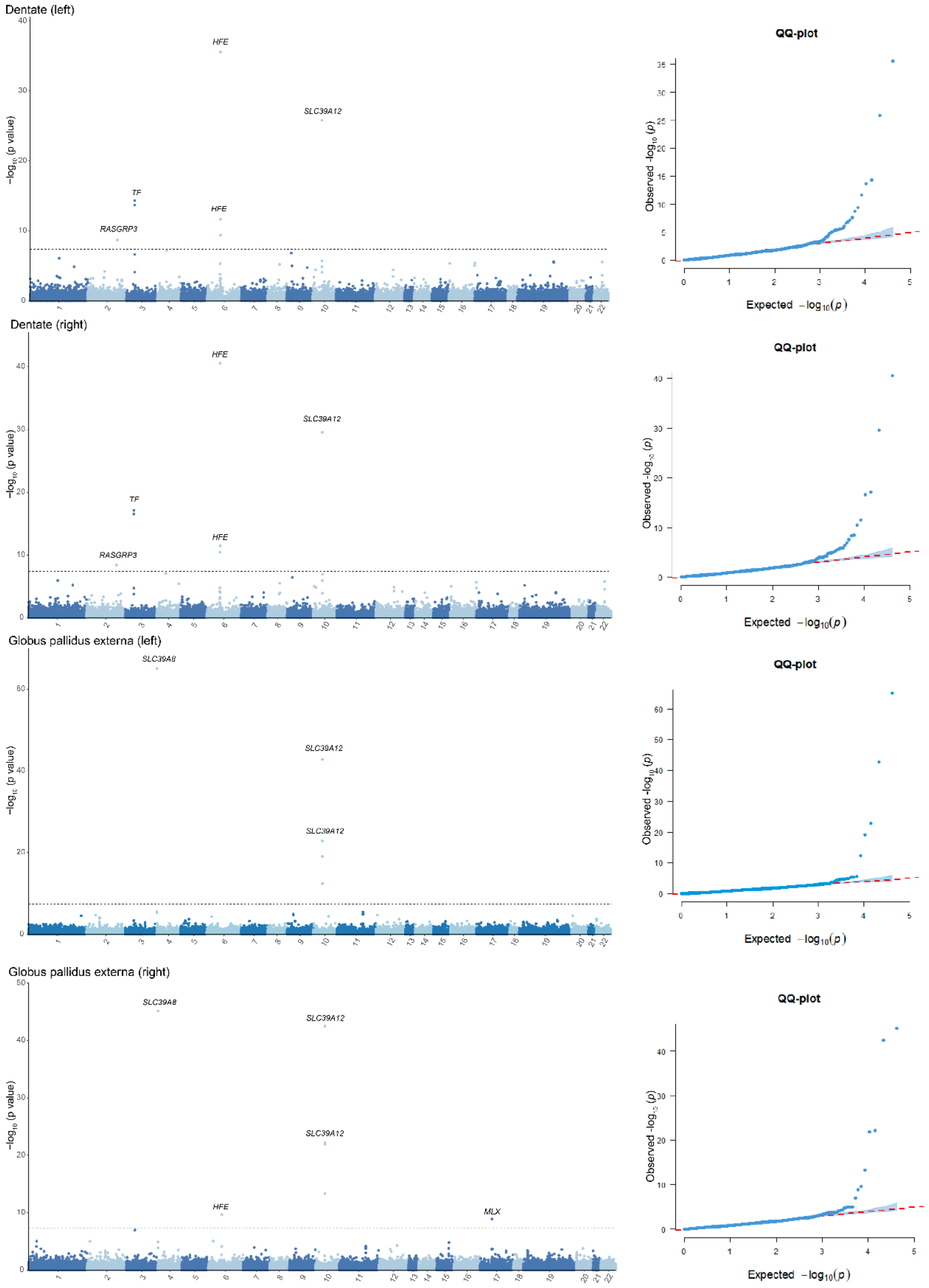
Dentate (left)
HFE
SLC39A12
TF
HFE
RASGRP3
−log10 (p value)
QQ-plot
Observed -log10 (p)
Expected −log10(p)
Dentate (right)
HFE
SLC39A12
TF
HFE
RASGRP3
−log10 (p value)
QQ-plot
Observed -log10 (p)
Expected −log10(p)
Globus pallidus externa (left)
SLC39A8
SLC39A12
SLC39A12
−log10 (p value)
QQ-plot
Observed -log10 (p)
Expected −log10(p)
Globus pallidus externa (right)
SLC39A8
SLC39A12
SLC39A12
HFE
MLX
−log10 (p value)
QQ-plot
Observed -log10 (p)
Expected −log10(p)

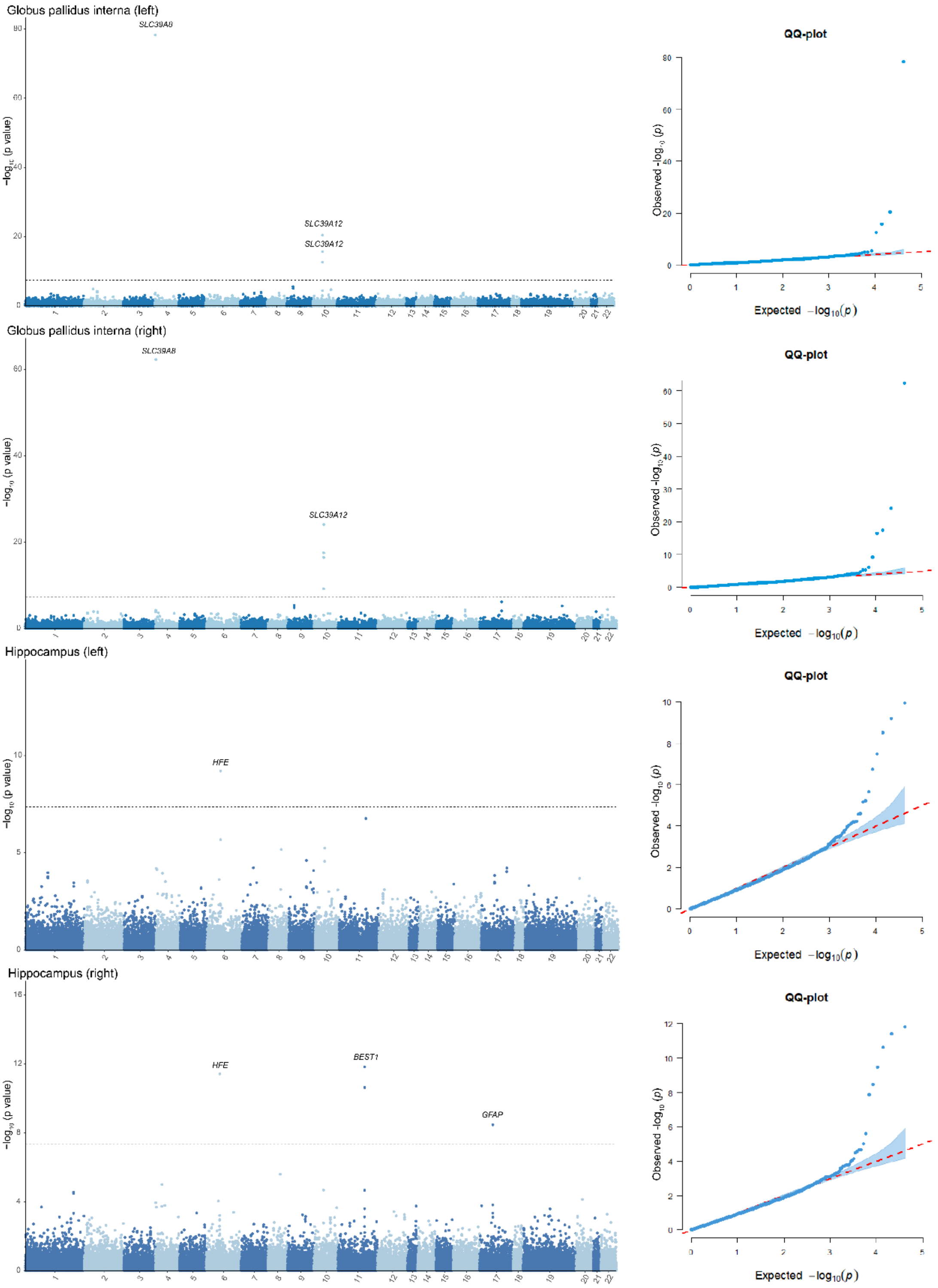

Globus pallidus interna (left)
SLC39A8
SLC39A12
SLC39A12
−log10 (p value)
QQ-plot
Observed -log10 (p)
Expected −log10(p)
Globus pallidus interna (right)
SLC39A8
SLC39A12
−log10 (p value)
QQ-plot
Observed -log10 (p)
Expected −log10(p)
Hippocampus (left)
HFE
−log10 (p value)
QQ-plot
Observed -log10 (p)
Expected −log10(p)
Hippocampus (right)
HFE
BEST1
GFAP
−log10 (p value)
QQ-plot
Observed -log10 (p)
Expected −log10(p)

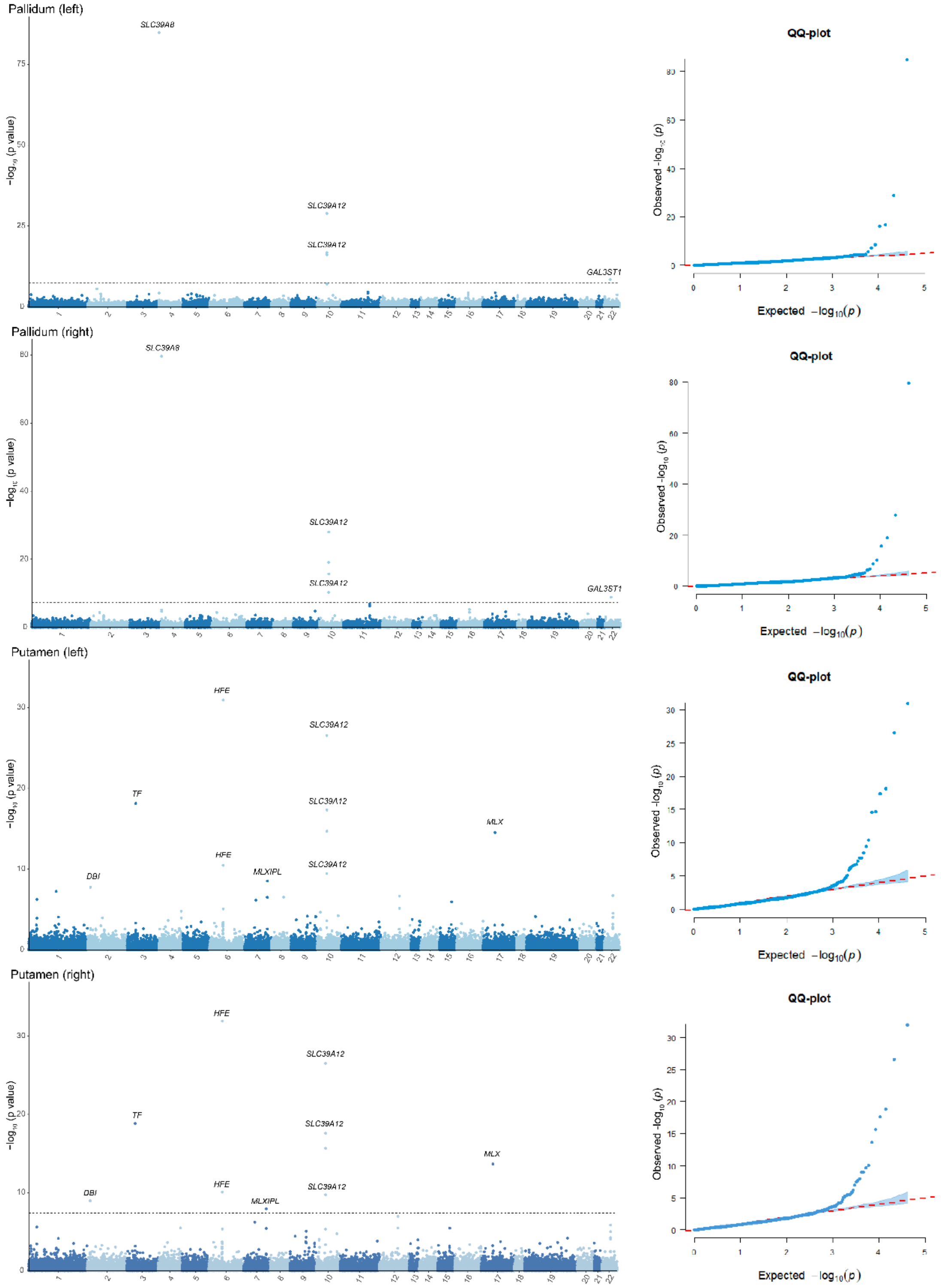
Pallidum (left)
SLC39A8
SLC39A12
SLC39A12
GAL3ST1
−log10 (p value)
QQ-plot
Observed −log10(p)
Expected −log10(p)
Pallidum (right)
SLC39A8
SLC39A12
SLC39A12
GAL3ST1
−log10 (p value)
QQ-plot
Observed −log10(p)
Expected −log10(p)
Putamen (left)
HFE
SLC39A12
TF
SLC39A12
MLX
HFE
SLC39A12
DBI
MLXIPL
−log10 (p value)
QQ-plot
Observed −log10(p)
Expected −log10(p)
Putamen (right)
HFE
SLC39A12
TF
SLC39A12
MLX
DBI
HFE
SLC39A12
MLXIPL
−log10 (p value)
QQ-plot
Observed −log10(p)
Expected −log10(p)

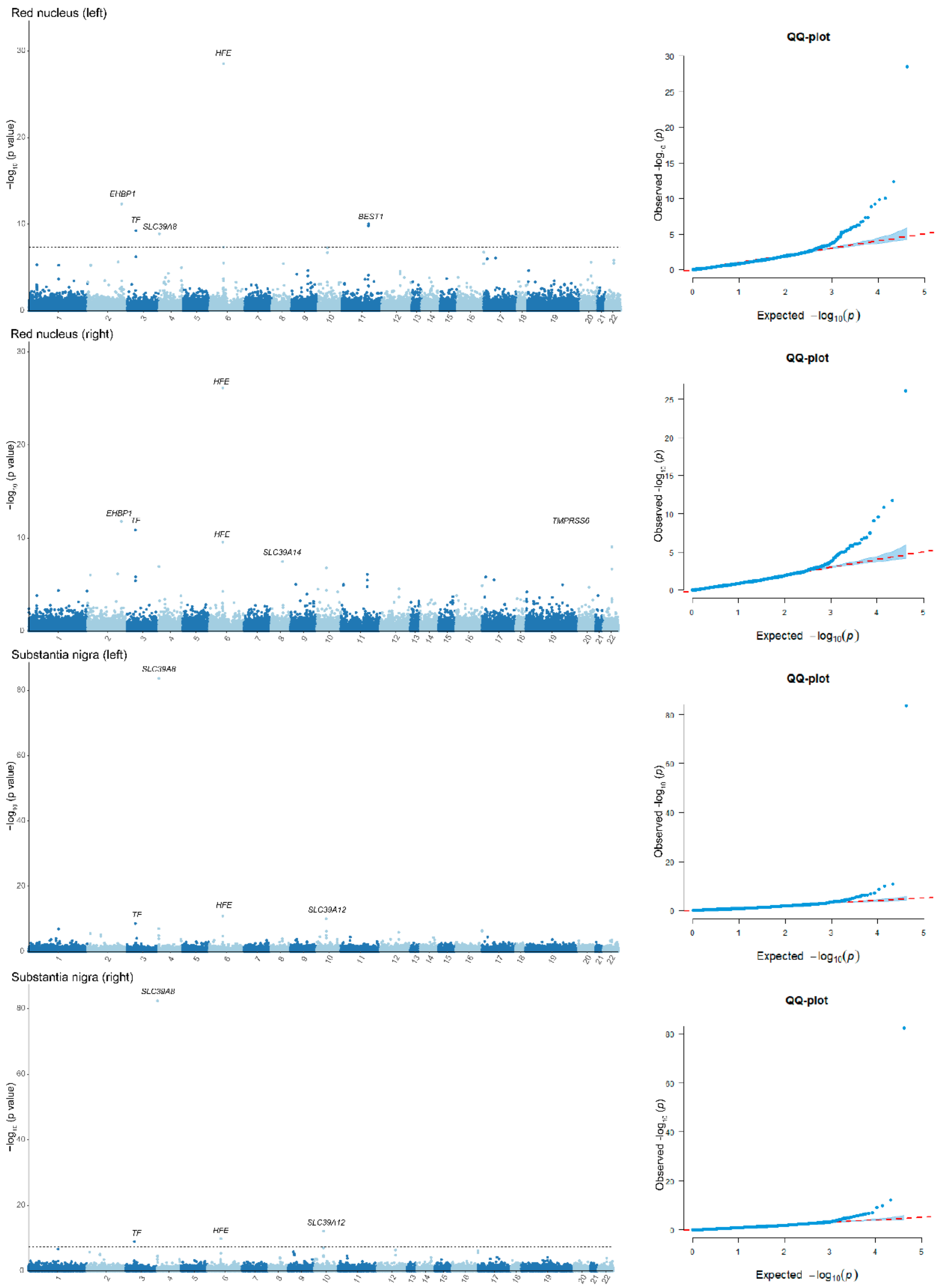

Red nucleus (left)
HFE
EHBP1
TF
SLC39A8
BEST1
QQ-plot
Red nucleus (right)
HFE
EHBP1
TF
HFE
SLC39A14
TMPRSS6
QQ-plot
Substantia nigra (left)
SLC39A8
TF
HFE
SLC39A12
QQ-plot
Substantia nigra (right)
SLC39A8
TF
HFE
SLC39A12
QQ-plot
$-\log_{10}$ (p value)
Observed $-\log_{10}(p)$
Expected $-\log_{10}(p)$

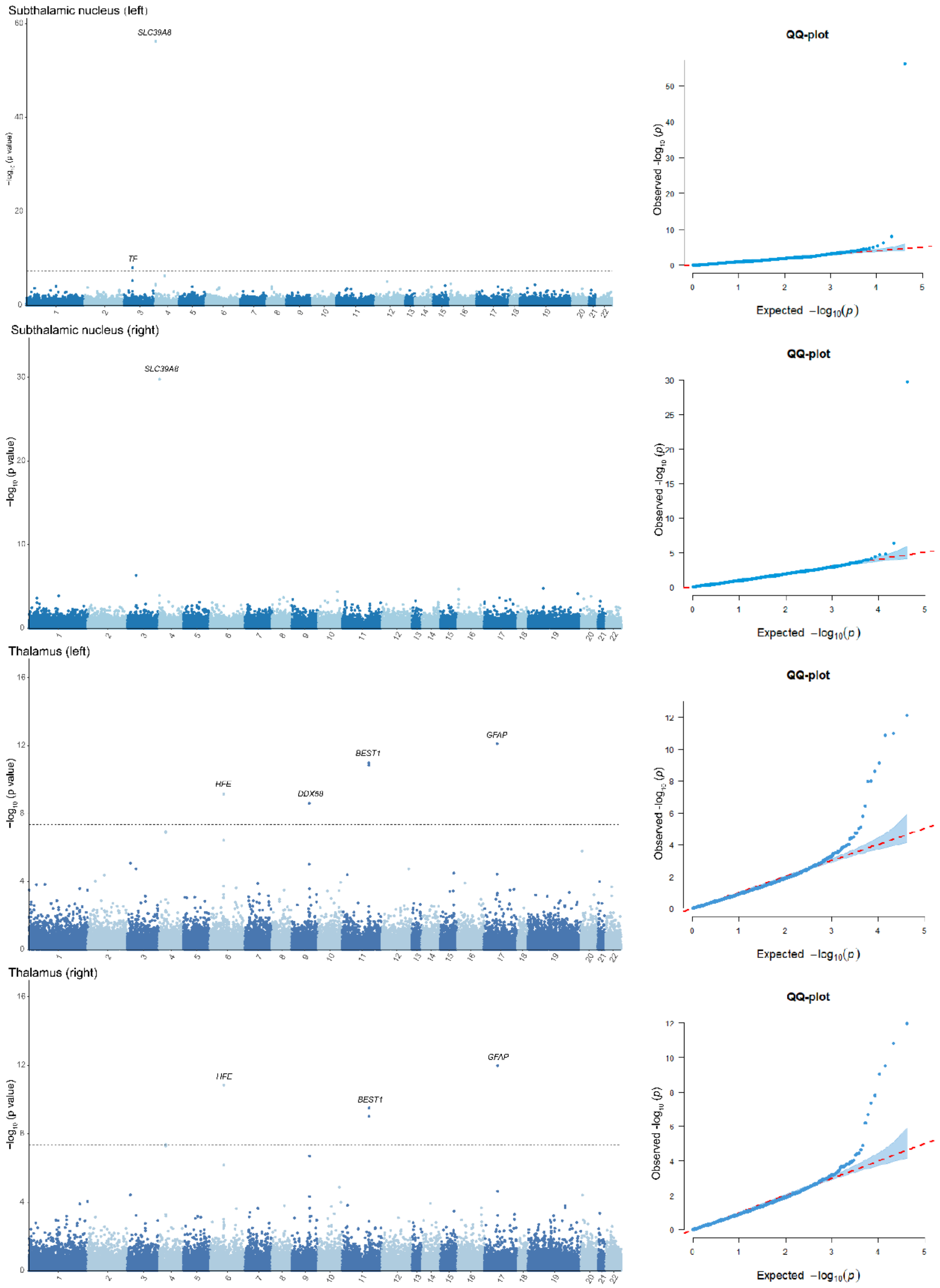

Supplementary Figure S2. Manhattan plots of every exome-wide association analysis of common protein-coding variants performed for QSM of 26 brain regions in 26789 participants. P values shown are two-sided and Bonferroni correction were used. The dotted horizontal line represents the significance threshold (4.6×10^{-8}).

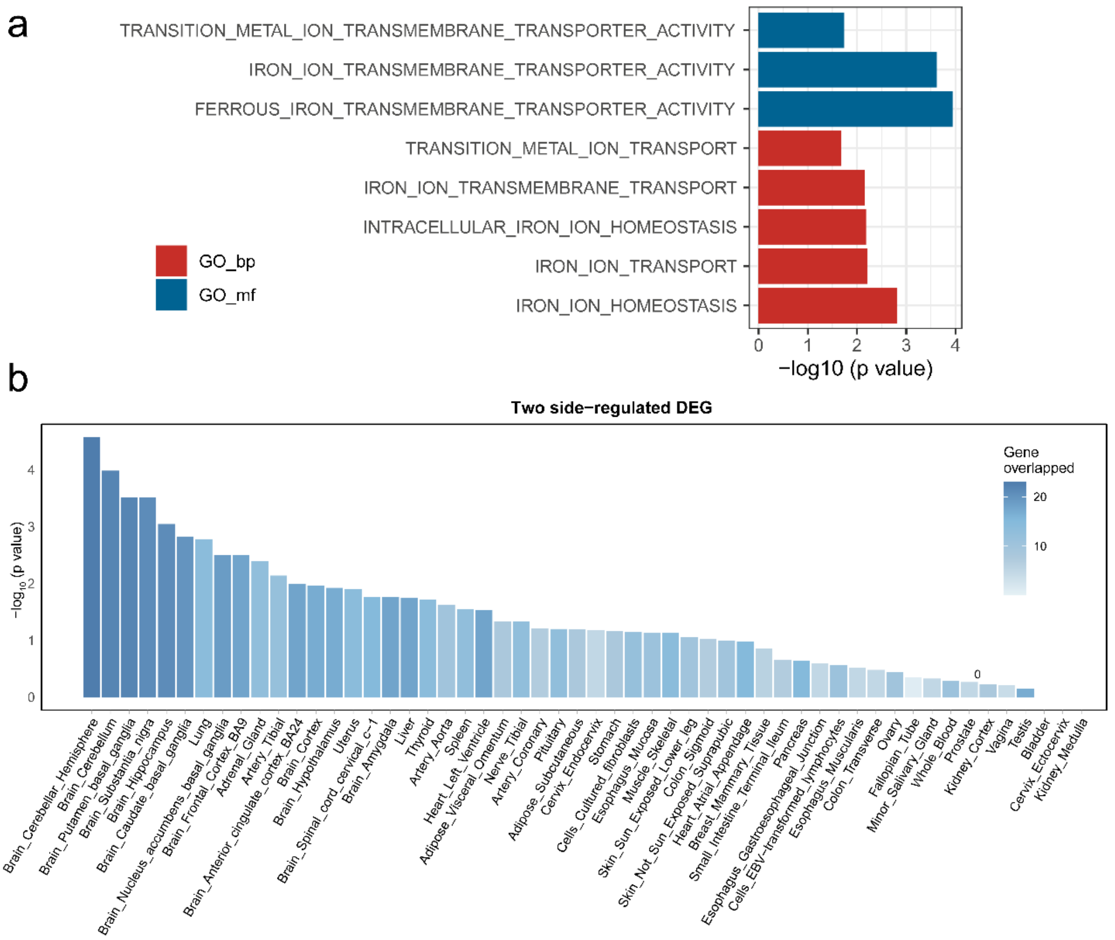

Supplementary Figure S3. Tissue and pathway enrichment analysis of genes (exclude top 5 genes) with brain iron. a) Gene set enrichment analysis of significant genes identified in the exome-wide association analysis, using Gene Ontology and KEGG Ontology database. b) Tissue-wide differential expression analysis of significant brain iron-related genes (exclude top 5 significant genes) using GTEx dataset. The p-values reported are two-sided and unadjusted. GOBP, Gene ontology biological process; GOMF, Gene ontology molecular function.
