# Supplementary material for: Whole-exome sequencing identifies protein-coding variants associated with brain iron in 29,828 individuals
Source: Nat Commun. 2024 Jul 2;15:5540. doi: 10.1038/s41467-024-49702-2 (PMC11219919; doi:10.1038/s41467-024-49702-2)
Supplement: Supplementary file 3 — Description of Additional Supplementary Files [file 41467_2024_49702_MOESM3_ESM.pdf]

Description of Additional Supplementary Files

File name: Supplementary Data 1
Description: Collapse analysis of significant rare variants of regional QSM (discovery)
File name: Supplementary Data 2
Description: Collapse analysis of rare variants of regional QSM (replication cohort)
File name: Supplementary Data 3
Description: Power calculation of cohorts
File name: Supplementary Data 4
Description: Single variants information and LOVO results for variants from significant Gene-regional QSM associations
File name: Supplementary Data 5
Description: Gene-regional QSM associations conditioned on nearby associated common variants
File name: Supplement Data 6
Description: Collapse analysis of rare variants of regional QSM in different sex.
File name: Supplementary Data 7
Description: Collapse analysis of significant common variants of regional QSM (discovery)
File name: Supplementary Data 8
Description: Genes mapped to common variants that associated regional QSM in replication study
File name: Supplementary Data 9
Description: Identified gene for brain iron that have previously been reported in GWAS of any traits listed in the GWAS catalog.
File name: Supplementary Data 10
Description: Gene set enrichment analysis of significant genes using Gene Ontology and KEGG Ontology database
File name: Supplementary Data 11
Description: Protein-protein interaction network of significant QSM genes
File name: Supplementary Data 12
Description: Tissue-wide differential expression analysis of significant QSM genes using GTEx dataset
File name: Supplementary Data 13
Description: Tissue-wide differential expression analysis of significant QSM genes (exclude top 5 genes) using GTEx dataset
File name: Supplementary Data 14
Description: Gene set enrichment analysis of significant genes (exclude top 5 genes) using Gene Ontology and KEGG Ontology database
File name: Supplementary Data 15
Description: Causal relationships identified by Mendelian randomization analysis.
File name: Supplementary Data 16
Description: Causal relationships identified by Mendelian randomization analysis

using other methods.

File name: Supplementary Data 17

Description: Inverse causal relationships identified by Mendelian randomization analysis.

File name: Supplementary Data 18

Description: Inverse causal relationships identified by Mendelian randomization analysis using other methods.

File name: Supplementary Data 19

Description: Causal relationships identified by multivariate Mendelian randomization analysis.

File name: Supplementary Data 20

Description: Field ID and description for regional QSM in the UK Biobank.
