# Supplementary material for: Whole-exome sequencing identifies protein-coding variants associated with brain iron in 29,828 individuals
Source: Nat Commun. 2024 Jul 2;15:5540. doi: 10.1038/s41467-024-49702-2 (PMC11219919; doi:10.1038/s41467-024-49702-2)
Supplement: Supplementary file 5 — Reporting Summary [file 41467_2024_49702_MOESM5_ESM.pdf]

nature portfolio

Corresponding author(s): Jin-Tai Yu, Wei Cheng

Last updated by author(s): May 9, 2024

# Reporting Summary

Nature Portfolio wishes to improve the reproducibility of the work that we publish. This form provides structure for consistency and transparency in reporting. For further information on Nature Portfolio policies, see our Editorial Policies and the Editorial Policy Checklist.

## Statistics

For all statistical analyses, confirm that the following items are present in the figure legend, table legend, main text, or Methods section.

| n/a | Confirmed | |
|---|---|---|
| ☐ | ☒ | The exact sample size (*n*) for each experimental group/condition, given as a discrete number and unit of measurement |
| ☐ | ☒ | A statement on whether measurements were taken from distinct samples or whether the same sample was measured repeatedly |
| ☐ | ☒ | The statistical test(s) used AND whether they are one- or two-sided<br>*Only common tests should be described solely by name; describe more complex techniques in the Methods section.* |
| ☐ | ☒ | A description of all covariates tested |
| ☐ | ☒ | A description of any assumptions or corrections, such as tests of normality and adjustment for multiple comparisons |
| ☐ | ☒ | A full description of the statistical parameters including central tendency (e.g. means) or other basic estimates (e.g. regression coefficient) AND variation (e.g. standard deviation) or associated estimates of uncertainty (e.g. confidence intervals) |
| ☐ | ☒ | For null hypothesis testing, the test statistic (e.g. *F*, *t*, *r*) with confidence intervals, effect sizes, degrees of freedom and *P* value noted<br>*Give P values as exact values whenever suitable.* |
| ☒ | ☐ | For Bayesian analysis, information on the choice of priors and Markov chain Monte Carlo settings |
| ☒ | ☐ | For hierarchical and complex designs, identification of the appropriate level for tests and full reporting of outcomes |
| ☐ | ☒ | Estimates of effect sizes (e.g. Cohen's *d*, Pearson's *r*), indicating how they were calculated |

*Our web collection on statistics for biologists contains articles on many of the points above.*

## Software and code

Policy information about availability of computer code

| | |
|---|---|
| Data collection | No software was involved in data collection (data used is all directly available from UK Biobank, as described in detail in the paper) |
| Data analysis | The following software and packages were used for data analysis: FUMA v.1.3.8 (https://fuma.ctglab.nl/), SnpEff (https://pcingola.github.io/SnpEff/), SAIGE-GENE+ (https://github.com/saigegit/SAIGE), PLINK (https://www.cog-genomics.org/plink/) and R v.4.2.0 (https://www.r-project.org/). The scripts used to conduct the main analyses are available at https://github.com/weikanggong/BrainIronWES. |

For manuscripts utilizing custom algorithms or software that are central to the research but not yet described in published literature, software must be made available to editors and reviewers. We strongly encourage code deposition in a community repository (e.g. GitHub). See the Nature Portfolio guidelines for submitting code & software for further information.

## Data

Policy information about availability of data

All manuscripts must include a data availability statement. This statement should provide the following information, where applicable:

- Accession codes, unique identifiers, or web links for publicly available datasets
- A description of any restrictions on data availability
- For clinical datasets or third party data, please ensure that the statement adheres to our policy

The UK Biobank brain imaging data of 29,828 participants (and 3,039 participants' repeat scan imaging data) were used in this study.
Permission to use the UK Biobank Resource was obtained via Material Transfer Agreement (https://www.ukbiobank.ac.uk/media/yfob3gln/access_031_f-

applicantmta-
data-only-v1-1.pdf) as part of Data Access Application 19542. All imaging data (including raw images, derived maps and IDPs), phenotypes and genetics data are made available by UK Biobank via their standard data access procedure (see http://www.ukbiobank.ac.uk/register-apply). Information on average time from application submission to data release can be found at https://www.ukbiobank.ac.uk/enable-your-research/apply-for-access.

## Research involving human participants, their data, or biological material

Policy information about studies with human participants or human data. See also policy information about sex, gender (identity/presentation), and sexual orientation and race, ethnicity and racism.

| | |
|---|---|
| Reporting on sex and gender | We took sex into considerations in our study and our findings could apply to both male and female. Sex in the UK Biobank was determined based on self-reporting data via questionnaire, and all included participants gave written informed consent for sharing of individual-level data. |
| Reporting on race, ethnicity, or other socially relevant groupings | Ethnic background (Field ID 21000, https://biobank.ctsu.ox.ac.uk/showcase/field.cgi?id=21000) was used to define ethnic backgrounds in this study. We included the British white participants in the main analysis. |
| Population characteristics | A total of 29,828 individuals of white British ethnicity were included in the WES analysis, aged between 40 to 69, and approximately 52% of whom were females. |
| Recruitment | The UKB enrolled the participants aged 40-69 years between 2006 and 2010 for baseline assessments in 22 centers across the UK. The assessment visits comprised interviews and questionnaires covering lifestyles and health conditions, physical measures, biological samples, imaging, and genotyping. The database is linked to national health datasets, including primary care, hospital inpatient, death, and cancer registration data. |
| Ethics oversight | UK Biobank has received ethical approval from the North West Multi-centre Research Ethics Committee (MREC, https://www.ukbiobank.ac.uk/learn-more-about-uk-biobank/about-us/ethics), and informed consent through electronic signature was obtained from study participants. This study utilized the UK Biobank Resource under application number 19542. |

Note that full information on the approval of the study protocol must also be provided in the manuscript.

# Field-specific reporting

Please select the one below that is the best fit for your research. If you are not sure, read the appropriate sections before making your selection.

☒ Life sciences ☐ Behavioural & social sciences ☐ Ecological, evolutionary & environmental sciences

For a reference copy of the document with all sections, see nature.com/documents/nr-reporting-summary-flat.pdf

# Life sciences study design

All studies must disclose on these points even when the disclosure is negative.

| | |
|---|---|
| Sample size | No statistical methods were used to predetermine sample sizes. All currently available sample in the UK Biobank were included. |
| Data exclusions | Participants without whole-exome sequencing data, those failed to pass quality control, individuals were excluded. |
| Replication | In this study, 3,039 participants with repeated scan brain imaging data formed the replication set. Statistical significance in the replication set was set at an uncorrected p-value threshold of <0.05. Of the thirty-six identified genes, sixteen can be replicated in replication set. |
| Randomization | There is nothing in this study that pertains to randomization. We are using existing data released by UK Biobank. UK Biobank is an observational prospective epidemiological study, and our study use all available subjects that fulfill the criteria described above. Hence there is no equivalent process of randomization that comes into this analysis (this is not a controlled randomised study). |
| Blinding | Blinding was not applicable to this study as this study is observational. |

# Reporting for specific materials, systems and methods

We require information from authors about some types of materials, experimental systems and methods used in many studies. Here, indicate whether each material, system or method listed is relevant to your study. If you are not sure if a list item applies to your research, read the appropriate section before selecting a response.

## Materials & experimental systems

| n/a | Involved in the study |
|---|---|
| ☒ | ☐ Antibodies |
| ☒ | ☐ Eukaryotic cell lines |
| ☒ | ☐ Palaeontology and archaeology |
| ☒ | ☐ Animals and other organisms |
| ☒ | ☐ Clinical data |
| ☒ | ☐ Dual use research of concern |
| ☒ | ☐ Plants |

## Methods

| n/a | Involved in the study |
|---|---|
| ☒ | ☐ ChIP-seq |
| ☒ | ☐ Flow cytometry |
| ☐ | ☒ MRI-based neuroimaging |

# Plants

Seed stocks

*Report on the source of all seed stocks or other plant material used. If applicable, state the seed stock centre and catalogue number. If plant specimens were collected from the field, describe the collection location, date and sampling procedures.*

Novel plant genotypes

*Describe the methods by which all novel plant genotypes were produced. This includes those generated by transgenic approaches, gene editing, chemical/radiation-based mutagenesis and hybridization. For transgenic lines, describe the transformation method, the number of independent lines analyzed and the generation upon which experiments were performed. For gene-edited lines, describe the editor used, the endogenous sequence targeted for editing, the targeting guide RNA sequence (if applicable) and how the editor was applied.*

Authentication

*Describe any authentication procedures for each seed stock used or novel genotype generated. Describe any experiments used to assess the effect of a mutation and, where applicable, how potential secondary effects (e.g. second site T-DNA insertions, mosiacism, off-target gene editing) were examined.*

# Magnetic resonance imaging

## Experimental design

Design type

Exome-wide association analysis of brain iron measured by quantitative susceptibility mapping derived from susceptibility-weighted MRI.

Design specifications

Not applicable, as our analyses did not use any functional MRI data.

Behavioral performance measures

Behavioral performance in the MRI scanner was not used in this study.

## Acquisition

Imaging type(s)

Susceptibility-weighted MRI

Field strength

3T

Sequence & imaging parameters

Susceptibility-weighted MRI: 3D dual-echo gradient echo (GRE) sequence
TE1=9.4ms, TE2=20ms, TE=27ms and an in-plane acceleration factor of 2
Field of view: 256x288x48 matrix
Resolution: 0.8x0.8x3.0 mm

Area of acquisition

Whole brain

Diffusion MRI ☐ Used ☒ Not used

## Preprocessing

Preprocessing software

Preprocessing of susceptibility-weighted MRI data were described in Wang et al., 2022.

Normalization

Quantitative susceptibility mapping (QSM) spatial maps were transformed using FNIRT (part of FSL) to a 1-mm T1 template in MNI-space, transformations were provided by the UK Biobank brain processing pipeline (as described in Alfaro-Almagro et al., 2018).

Normalization template

MNI152 1mm standard space.

Noise and artifact removal

Noise and artifact removal was performed to susceptibility-weighted MRI data based on Wang et al., 2022.

Volume censoring

No volume censoring was performed to susceptibility-weighted MRI data.

## Statistical modeling & inference

Model type and settings: Exome-wide association studies with multiple comparison correction (Bonferroni) in subcortical and cerebellar structures.

Effect(s) tested: Not applicable

Specify type of analysis: ☐ Whole brain ☒ ROI-based ☐ Both

Anatomical location(s): Deep grey matter structures (accumbens, amygdala, caudate, hippocampus, pallidum, putamen and thalamus) segmented using FIRST (as part of central UKB release), substantia nigra,red nucleus, subthalamic nucleus, globus pallidus externa, globus pallidus interna and dentate segmented using multi-contrast PD25 atlas and the deep cerebellar nuclei probabilistic atlas.

Statistic type for inference (See Eklund et al. 2016): Not applicable

Correction: Bonferroni correction at P corrected < 0.05.

## Models & analysis

| n/a | Involved in the study |
| --- | --- |
| ☒ | ☐ Functional and/or effective connectivity |
| ☒ | ☐ Graph analysis |
| ☒ | ☐ Multivariate modeling or predictive analysis |
